# The FiCTION dental trial protocol – filling children’s teeth: indicated or not?

**DOI:** 10.1186/1472-6831-13-25

**Published:** 2013-06-01

**Authors:** Nicola PT Innes, Jan E Clarkson, Chris Speed, Gail VA Douglas, Anne Maguire

**Affiliations:** 1 Dundee Dental Hospital and School, University of Dundee, Park Place, Dundee DD1 4HN, UK; 2 Newcastle Clinical Trials Unit, Institute of Health and Society, Newcastle University, 4th Floor William Leech Building, Framlington Place, Newcastle upon Tyne NE2 4HH, UK; 3 Dental Institute, Leeds University, Clarendon Way, Leeds LS2 9LU, UK; 4 Centre for Oral Health Research, School of Dental Sciences, Newcastle University, Framlington Place, Newcastle upon Tyne NE2 4BW, UK

**Keywords:** Dental caries, Caries prevention, Primary teeth, Prevention, Paediatric Dentistry, Restoration, Fillings, RCT, Primary care

## Abstract

**Background:**

There is a lack of evidence for effective management of dental caries (decay) in children’s primary (baby) teeth and an apparent failure of conventional dental restorations (fillings) to prevent dental pain and infection for UK children in Primary Care. UK dental schools’ teaching has been based on British Society of Paediatric Dentistry guidance which recommends that caries in primary teeth should be removed and a restoration placed. However, the evidence base for this is limited in volume and quality, and comes from studies conducted in either secondary care or specialist practices. Restorations provided in specialist environments can be effective but the generalisability of this evidence to Primary Care has been questioned.

The FiCTION trial addresses the Health Technology Assessment (HTA) Programme’s commissioning brief and research question “What is the clinical and cost effectiveness of restoration caries in primary teeth, compared to no treatment?” It compares conventional restorations with an intermediate treatment strategy based on the biological (sealing-in) management of caries and with no restorations.

**Methods/Design:**

This is a Primary Care-based multi-centre, three-arm, parallel group, patient-randomised controlled trial. Practitioners are recruiting 1461 children, (3–7 years) with at least one primary molar tooth where caries extends into dentine. Children are randomized and treated according to one of three treatment approaches; conventional caries management with best practice prevention, biological management of caries with best practice prevention or best practice prevention alone.

Baseline measures and outcome data (at review/treatment during three year follow-up) are assessed through direct reporting, clinical examination including blinded radiograph assessment, and child/parent questionnaires.

The primary outcome measure is the incidence of either pain or infection related to dental caries.

Secondary outcomes are; incidence of caries in primary and permanent teeth, patient quality of life, cost-effectiveness, acceptability of treatment strategies to patients and parents and their experiences, and dentists’ preferences.

**Discussion:**

FiCTION will provide evidence for the most clinically-effective and cost-effective approach to managing caries in children’s primary teeth in Primary Care. This will support general dental practitioners in treatment decision making for child patients to minimize pain and infection in primary teeth. The trial is currently recruiting patients.

**Trial registration:**

Protocol ID: NCTU: ISRCTN77044005

## Background

The lack of evidence for the effective management of dental caries (decay) in children’s primary teeth is causing considerable uncertainty for the dental profession and patients. In particular, the apparent failure of current practice in UK Primary Dental Care to prevent pain and infection in children [1] has prompted much debate. Teaching in UK dental schools is based on guidance from the British Society of Paediatric Dentistry (BSPD) which includes the recommendation that the optimum treatment of caries in primary teeth should be its removal, followed by the placement of a conventional restoration (filling) to replace lost tooth tissue [2,3]. However, these recommendations are largely based on evidence for the effectiveness of restorations obtained from studies conducted in either a secondary care or specialist paediatric dental practice setting [4].

Within the United Kingdom (UK), the majority of dental care for children is carried out in Primary Care by general dental practitioners (GDPs) who currently provide care under different funding systems for general dental services. In Scotland, the capitation and fee per item of service system is in operation and to assist healthcare workers and patients the Scottish Dental Clinical Effectiveness Programme has developed national guidance for the management of caries in children [5]. In England and Wales, many Primary Care Trusts (PCTs) are now seeking to secure adherence to best practice guidance as part of their clinical governance responsibilities when commissioning dental Primary Care services. Whilst the implication of the funding systems for the type and quality of care is unknown, there is universal agreement that guidance for the effective management of caries is needed.

It is acknowledged that restorations provided in specialist clinical environments can be effective, however, both the volume and quality of the research on which current guidance is based is limited [6]. Moreover, the generalisability of this evidence to a Primary Care setting has been questioned although there is supportive evidence for a restoration-based approach to managing decay in primary teeth in this environment which comes from a study of outcomes based on dental survey/record-based survival data [7]. Perhaps the perceived ineffectiveness of the traditional “drill and fill” methods of managing decayed primary teeth is one reason this approach is not popular with GDPs [8]. Less than 13% and 14% of decayed teeth in 5 year-old children in Scotland and England respectively are currently filled [9,10]. The lack of direct evidence relevant to the setting where the vast majority of children are seen, i.e. general dental practice, and the discrepancy between the evidence for restorative management of caries in the primary and secondary care settings, complicate the refinement of the process of care for what is the most common disease of young children.

Three recent studies, conducted in general dental practice in the UK, have provoked the current debate around appropriate and effective dental care for children with caries in primary teeth. The first of these was a retrospective case note study, based on a group of 50 GDPs’ patient records, which suggested that placing a restoration, compared with leaving the tooth unfilled, did not improve the clinical outcome in terms of dental pain and infection [1]. In fact, the likelihood of children with filled teeth experiencing dental pain or infection was similar to that reported for the second study of 481 children who attended two related general dental practices with a practice policy of leaving asymptomatic carious primary teeth unrestored, focussing on a preventive strategy alone to manage them [6]. The third, and most recent study, was a randomised controlled trial involving 18 GDPs. The results demonstrate the ineffectiveness of a conventional approach (that is drilling out caries and placing a restoration) to treating caries in children in general dental practice. This trial showed a failure rate in terms of pain and infection, after two years, approaching that reported by the previous two studies for unrestored teeth [11] and the high failure rates continued at five year follow up [12].

A Cochrane review [13], first published around the time this protocol was developed, found emerging biologically orientated strategies for managing caries (sealing some of the decay within the tooth rather than drilling it all out) to be effective. An update of that review has confirmed this finding with further trials. In addition, a “biological” method of managing primary teeth by sealing in the caries with preformed metal crowns (PMCs) has been found, in a trial set in general dental practice, to be both effective at preventing pain and infection, and acceptable to children, parents and GDPs. The follow up results at five years compared favourably with conventional restorations when carried out in secondary care and private practice [11,12].

There is a gulf between the management strategies for decayed primary teeth taught in UK dental schools and the treatment currently being provided by GDPs. These management strategies can be grouped into three: 1) the conventional approach (commonly known as the ‘drill and fill’ method) which is the traditional approach to managing caries that has been taught and practiced for many years and is based on active management of caries by its complete removal and placement of a traditional restoration or preformed metal crown; 2) the biological approach where caries is sealed into the tooth, separating it from the oral environment to slow or stop its progression using adhesive restorations or preformed metal crowns, or; 3) best practice prevention alone which is aimed at slowing down the rate of tooth decay and is where no caries removal, restoration or sealing-in caries is carried out. As yet, there is insufficient evidence upon which to base a recommendation on which of these three management strategies is the most effective at managing dental caries in children treated in Primary Care. The implication of this research is likely to be a change in policy for service and education in the NHS and beyond.

### Trial purpose and objectives

The primary objective is to compare the incidence of pain and infection experienced over a period of three years in 3–7 year-old children with caries in primary teeth when managed by one of these three treatment strategies.

The secondary objectives are to compare the three treatment strategies with respect to: incidence of caries in primary and secondary teeth, patient quality of life, cost-effectiveness over the period of the study, acceptability and associated experiences for patients and parents, and dentists’ preferences.

#### Research ethics approval

The conduct of this study will be in accordance with the ethical principles set out in the Declaration of Helsinki (2008) and the principles of Good Clinical Practice in line with the Research Governance Framework for Health and Social Care [14].

A favourable ethical opinion from the East of Scotland Research Ethics Service was sought and obtained on the 24th July 2012 (12/ES/0047). In addition, local Research and Development approval was sought from each NHS Trust and Health Board for each participating site prior to commencement of the study.

## Methods/design

### Basis for the study design and setting

The trial is set in Primary Care, reflecting the setting within which the vast majority of children’s dentistry is carried out and arranged around five clinical centres in the UK; Dundee/Glasgow, Newcastle, Sheffield/Leeds, London and Cardiff. The results of the FiCTION Pilot Rehearsal Trial (Protocol ID: NCTU:ISRCTN77044005) and the parallel FiCTION Feasibility Study (Protocol ID: NCTU:FS77044005), which were carried out between 01/01/10 and 31/10/11 have been reported to the HTA. In addition, findings from qualitative interviews with dentists, child participants and their parents on their views of participation in the Rehearsal Trial [15] have been used to inform improvements in the conduct and minor refinement to the design of the trial.

### Practices

#### *Target sample size*

The primary outcome will be the proportion of children reporting either pain or infection during the three years of follow up. Based on evidence from previous studies on similar populations with no restorations [1,6] and conventional restorations and the Hall Technique [11], infection rates of 20%, 10% and 3% respectively are expected. Using the “sampsi” procedure (a sample size calculation based on a two-sample test of proportions assuming a normal approximation and incorporating a continuity correction) in Stata version 9 [16], and assuming a significance level of 2.5% (to allow for multiple testing involved in a three arm trial) the following are needed:

● two groups of 334 children to detect a difference in rates between 10% and 20% with 90% power

● two groups of 334 children to detect a difference in rates between 3% and 10% with 90% power

The sample size has been increased by an arbitrary inflation factor of 1.09 (giving 365 children per arm at end of follow up) to allow for adjustment of estimates of effect size taking into account variation between randomisation strata (dental practices). Allowing for a loss to follow up over three years of 25% (based on experience other clinical trials in Primary Care, including the FiCTION Pilot Trial), we will need to randomise 487 children to each arm of the trial.

**Figure 1:**
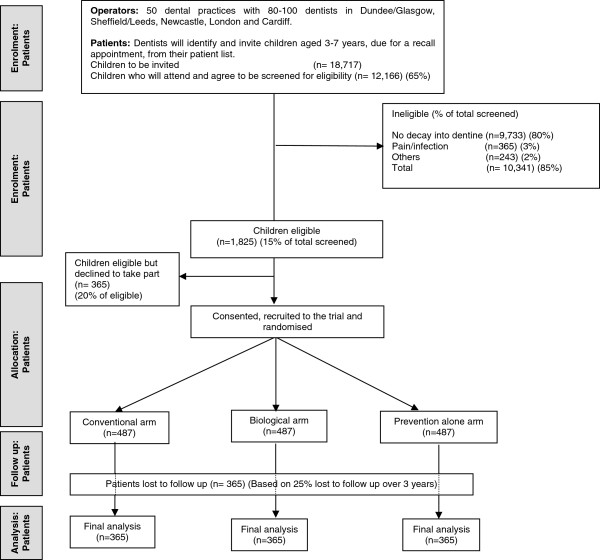
FiCTION Trial flow diagram showing projected numbers of participants throughout trial.

We are aiming to recruit 50 practices with a total of 80–100 dentists. Based on the findings from the Pilot Rehearsal Trial, from these fifty practices 18,717 children will be invited to attend for screening with an expected 12,166 (65%) of these children actually attending and agreeing to be screened for the study. It is expected that 1825 children (15% of those screened) will be eligible for the Trial. Of these, it is anticipated that 1460 (80% of those eligible) will consent to be randomised, with 487 children allocated to each of the three study arms. Figure 1 shows projected numbers of participants in the trial in a Consort flow diagram.

#### *Eligibility*

Practices will be eligible for participation in the study if they:

● see and treat children aged 3–7 under NHS contracts;

● see children with dental caries in primary teeth (around 1 child per week is considered an appropriate frequency for recruitment rate purposes); and

● have the infrastructure to support the study i.e. electronic (or accessible non-electronic) patient management systems and internet access.

#### *Recruitment and retention*

The fifty practices (approximately ten from each of the five centres; Dundee/Glasgow, Newcastle, Sheffield/Leeds, London and Cardiff) will comprise around 80–100 dentists and their dental teams. Selection of these practices will reflect the socio-demographic mix of the catchment communities. Our strategies to recruit and retain practices are both generally and locally targeted. These have been developed by the trial team in conjunction with the local Clinical Leads and their Trial Assistants. Following an initial information letter, practices expressing interest in participating in the trial were visited by the research team to assess their eligibility before being given a formal invitation to participate.

**General strategy** Practices invited to participate are those which:

● participated in the Pilot Rehearsal Trial in Scotland, Newcastle and Sheffield Clinical Centres (n=13);

● had been contacted as part of the Feasibility Study (60 randomly selected practices in each of 4 areas and 33 in one area; n=273) and responded expressing interest in participating (n=70);

● had formed part of the sampling frame for the feasibility study but were NOT sampled as part of that study (n=632 in the 5 areas);

● responded to general advertising in the dental press, expressing an interest.

These practices will all be considered in accordance with practice eligibility criteria and proximity to the Clinical Centres.

**Local strategy** For each of the regions in the trial existing local research networks and a variety of formal and informal opportunities were used to engage with practices. Clinical Leads for each region have and will continue to develop tailored local strategies to enhance practice recruitment, running in parallel to the general strategy. This has comprised email and postal mailing of FiCTION flyers to practices and practitioners by Comprehensive Local Research Networks (CLRN) and some Primary Care Research Networks (PCRN), and their equivalents in Wales and Scotland. Expressions of interest were followed up locally by the Clinical Leads with the support of the local Primary Care research networks. Local practice recruitment meetings have been held to inform interested GDPs about the FiCTION Trial and answer any questions they may have. In addition, the trial team have attended other events where GDPs are present to raise awareness of the trial.

#### *Trial-specific clinical and trial process training*

Each Clinical Centre has hosted a Practice Training Day to deliver clinical and trial process training to all enrolled dentists and dental team staff. For dental team staff who cannot attend a Practice Training Day, training is being delivered as part of the Site Initiation Visit by the Trial Manager, Clinical Researcher and Clinical Lead.

Training was provided for individual clinical procedures and in treatment planning for dental care appropriate to each arm of the trial. This involved a didactic teaching session followed by practical treatment planning with cases and discussion with the local Clinical Lead and Chief Investigators. Training was also given in discussion of the trial with families and consent/assent procedures.

### Participants

**Figure 2:**
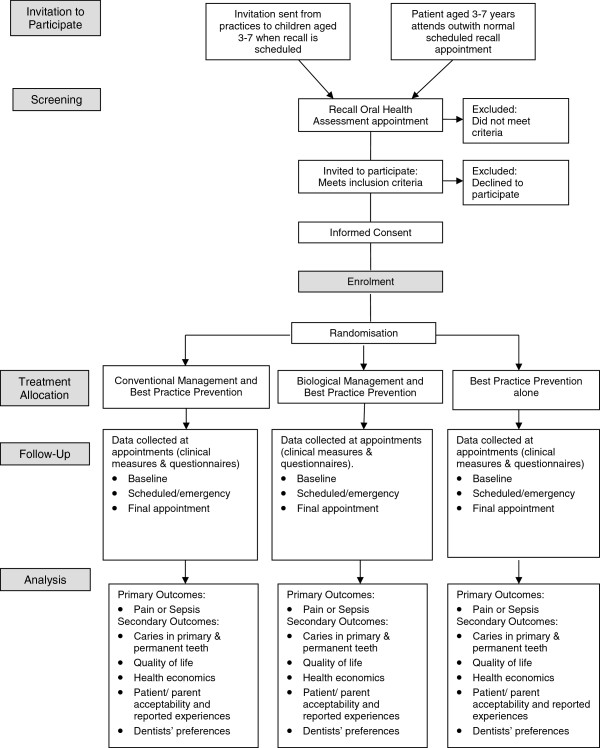
Screening, recruitment, randomisation and participant follow-up schedule for the FiCTION Trial.

Children within the correct age range will be identified through participating practices and a letter inviting them to take part, along with a parent and child version of the Patient Information Sheet, will be sent with their next check-up appointment. Eligible children may also be identified at a scheduled dental visit. In these cases information sheets are given to the family when they attend with a minimum period of 24 hours given to allow participation to be considered. Figure 2 shows the screening, recruitment, randomization and participant follow-up schedule.

#### *Inclusion criteria*

Child patients (3–7 years of age), who:

1. are willing to be dentally examined;

2. have at least one primary molar tooth with caries into dentine, and;

3. are known regular attendees or, if new to the practice, considered likely to return for follow-up.

#### *Exclusion criteria*

Patients:

1. accompanied by an adult who lacks the legal status or mental capacity to give informed consent;

2. who, at the recruitment appointment, present with either pain or dental infection (however, patients can be reconsidered for recruitment at a later appointment when they are pain and infection free);

3. with a medical condition requiring special considerations with their dental management;

4. currently involved in any other research which may impact upon this study, or;

5. who will be moving out of the catchment area for the dental practice during the 3 years following recruitment.

#### *Consent and assent*

Following their dental check-up and confirmation of eligibility, parents and children who are interested will have the trial discussed with them by their dentist or another trained person in the practice. The parent(s)/legal guardian(s) of all children in the study will provide written informed consent before any study procedures are carried out and a participant information sheet and parent information sheet will have been provided to facilitate this process. In so far as possible, and with the agreement of the parent(s)/legal guardian(s), participating children will also be asked to provide written or oral assent. Those not competent in English will be invited to bring an interpreter with them to the recall appointment or to request an NHS interpreter where this service is available.

Full training in taking informed consent in a paediatric setting was provided as part of the Practice Training day. A trained member of practice team and the child will sign and date the Assent form whilst the parent and trained staff member will sign the Informed Consent Form(s) to confirm that consent has been obtained. The participant will then receive a copy of the consent form and a copy will be filed in the Investigator Site File (ISF). The original will be kept in the patient’s records.

#### *Participant allocation and blinding*

The trial will comprise simple randomisation of patients into the three caries management strategies in a 1:1:1 ratio. Randomisation will be through the web-based, automated central randomisation facility at the Newcastle Clinical Trials Unit (NCTU) using variable length random permeated blocks to ensure concealment of allocation.

The different treatments that will be applied in each arm mean that it is not possible to blind the parents, children, or dentists as to which arm the child is participating in.

#### *Treatment of participants*

Following random allocation of the child to one of the three treatment strategies, FiCTION-trained dentists will plan treatment, manage and follow up the child according to allocation status. The intention is that patients will be managed throughout their time in the study according to the randomisation arm to which they were allocated, i.e. any subsequent episode of caries will be managed in the same way (as per random allocation) as the initial episode. Any crossover that does occur because patients or parents opt to have treatment that is part of another arm will be recorded and monitored. Similarly, if the clinician delivers treatment outwith the allocated arm, this will be recorded and monitored. At the treatment appointments, the parents and children will complete questionnaires. Participants will be followed up for three years and data on all treatment provided over the study period will be collected annually at a FiCTION recall visit.

#### *Withdrawal procedures*

Parent(s)/legal guardian(s) will be informed that they have the right to withdraw from the study at any time. The right to refuse to participate without giving reasons will be respected. After the participant has entered the study, the clinician will remain free to give alternative treatment to that specified in the protocol at any stage if he/she feels it is in the participant’s best interest, but the reasons for doing so will be recorded. In these cases the participants will remain within the study for the purposes of follow-up and data analysis. All participants will be free to withdraw at any time from the protocol treatment without giving reasons and without prejudicing further treatment.

There are two withdrawal options:

1. withdrawing completely (i.e. withdrawal from both the study treatment and provision of follow-up data); and

2. withdrawing partially (i.e. withdrawal from study treatment but continuing to provide follow-up data by attending the practice and completing questionnaires).

Consent will be sought from participants choosing Option 1 to retain data collected up to the point of withdrawal. Participants will be asked if they would be willing for the reason to withdraw to be recorded, as strong preferences for one of the three treatment arms may help inform public perceptions of the treatment options.

### Recruitment and retention strategy for practices and participants

The practice and participant retention strategy is based around actively maintaining contact with all trial practitioners throughout the study (there will be fortnightly emailed updates and regular newsletters), showing their time is valued (through CPD and remuneration). To help facilitate ease of access to trial relevant resources, such as electronic copies of the trial protocol, clinical protocols, participant information leaflets and frequently asked question documents a secure web-based virtual research environment is available which the practices have access to. Early identification of problems will allow us to work closely with the practitioners to troubleshoot. Active support for the practices and participants from the PCRNs, research networks and local research champions will also be sought and a final report will be issued to all participating dentists.

Each practice has a target of 30 children to recruit over a 12 month recruitment period. The first practice was ready to recruit on the 21st September 2012.

### Interventions

Three treatment strategies for managing caries in the primary dentition are being tested with each patient being allocated to one strategy and managed within that arm of the trial for three years.

### Conventional management of caries, with best practice prevention

Local anaesthesia (a dental injection) is placed to numb the tooth, dentinal caries is mechanically removed using rotary instruments (drill) or by hand excavation (using hand tools) and a restoration is placed in the tooth to fill the cavity. If the dental pulp is exposed during caries removal or there are symptoms of pulpitis, a pulpotomy (partial root treatment) may be carried out. Retained roots, and teeth for which the crowns are unrestorable or the pulp chamber is open, are managed by extraction (removal) of the tooth following local anaesthesia.

Best practice prevention is carried out in line with current guidelines and as described below.

### Biological management of caries, with best practice prevention

Dentinal caries is sealed into the tooth, and separated from the oral cavity by application of an adhesive restoration material over the decay, or by covering with a metal crown. Decay may, on occasion, be partially removed prior to the tooth being sealed. Injections are rarely needed. Retained roots, and teeth for which the crowns are unrestorable, or dental nerves (pulps) exposed with active caries (still progressing) or where the clinician decides the tooth is likely to cause the patient pain or infection before it exfoliates (falls out) are managed by extraction.

Best practice prevention is carried out in line with current guidelines and as described below.

### Best practice prevention alone

Dentists and other members of the dental team base patients’ treatment plans on best practice preventive care for teeth and oral health. This will involve four strands, all carried out according to current national guidelines [5,17,18]:

● Toothbrushing/self-applied topical fluoride use;

● Dietary investigation, analysis and intervention;

● Fissure sealants for secondary teeth; and,

● Fluoride varnish applied to primary and secondary teeth.

### Data collection, management and analysis

Outcome data is being collected through a clinician-completed Case Report Form (CRF) and via child and parent questionnaires. Table 1 details the scheduling of individual outcome data collection events, how each outcome is captured, by whom, and when.

**Table 1:** FiCTION Trial: Schedule of outcome data collection events

**Event**	**Completed by:**	**Baseline examination appointment**	**Treatment appointments (Scheduled treatment or recall & unscheduled/ emergency)**	**Final appointment at 3 years post randomisation**
Bitewing Radiographs	GDP	Risk-based in line with guidance		
		**NOT A STUDY INVESTIGATION**		
Consent/Assent & Randomisaton	GDP	X		
ICDAS (CRF)	GDP	X		X
Adverse Event recording (CRF)	GDP		X	X
Pain: post treatment questions to GDP (CRF)	GDP		X	X
Cooperation (CRF)	GDP		X	X
Intervention Cost data (CRF)	GDP		X	X
Discomfort during treatment (DDQ8)	Parent		X	X
Quality of Life	Parent	X		X
Worry and Pain pre/post treatment questions to parent	Parent		X	X
Economic questions	Parent		X	X
MCDAS & worry	Child	X	X	X
Pain: pre/post treatment questions to child: VAS	Child		X	X

### Primary outcomes

#### *Pain (toothache)*

Assessments for pain from toothache are be made at each visit (treatment or recall) throughout the patient’s participation in the trial using the Dental Discomfort Questionnaire (DDQ8) completed by the parents [19]. In order to differentiate between pain originating from a decayed tooth and pain from other causes, the dentist forms a diagnosis based on patient/parent history and the clinical evidence available from examination, which is recorded on the CRF. This outcome is the number of children in each treatment arm experiencing toothache pain and the number of episodes of pain for each child in each arm during the 3-year follow-up period.

#### *Dental infection*

Assessments for infection are made at each visit (treatment or recall) throughout the patient’s participation in the trial. The outcomes are clinical (from examination by the child’s dentist) and radiographic signs (assessed by a dentist and an independent assessor). Clinical visual examinations for infection are specifically undertaken at every dental visit by the GDPs, and recorded on the CRF. These examinations are supplemented with independent examination of any bitewing radiographs that have been taken in line with Faculty of General Dental Practitioners guidelines [20] to record radiographic signs of inter-radicular pathology. The clinical detection criteria for the positive recording of infection are the presence of a swelling, dental abscess or draining sinus. Although GDPs are familiar with the signs and symptoms of infection we have developed the FiCTION training programme to specifically include the detection of infection and ensure it is reliably and reproducibly recorded.

Data for the primary outcomes of pain and infection are recorded during or following appointment times when the participant attends for both scheduled appointments and unscheduled/emergency appointments.

### Secondary outcomes

#### *Incidence of caries in primary and secondary teeth*

Detailed measurements of caries experience are recorded at baseline and final assessment by the GDPs using the CRF. The dentists measure both early and more advanced stages of dental caries. The stage or extent of caries is recorded using the International Caries Detection and Assessment System [21] which the participating dentists have received training in. The primary requirement for the examination is clean, dry teeth. All surfaces of all teeth are examined and the status of each recorded in terms of caries and restorations. Bitewing radiographs, taken in line with FGDP (UK) guidelines [20] (with blinded, independent assessment) are used as an independent measure of dental caries. However, as frequency of bitewing radiographs is based on caries risk assessment, and as some children may move out of the high risk group during the course of the trial, the frequency of bitewing radiographs taken for some children may reduce over the period of the study.

#### *Quality of Life*

Oral health related quality of life is measured at the beginning and end of the study. The measurement of quality of life in children is complicated by the rapid changes seen as children grow [22,23] including the development of children’s levels of literacy and understanding. For children under six years of age, the use of simple child-completed scales or questionnaires completed by parents as proxies is the usual solution [24].

Parents will be asked to complete a 16-item Parents’ Perception Questionnaire (PPQ) (Murray Thomson personal communication. OHRQoL Symposium, BSODR, Sheffield, 2011). The full length version of this measure has been found to be reliable and valid for use in the UK [25]. In addition, parents will be asked to provide a proxy evaluation of their child’s overall oral health-related quality of life by responding to two single item ratings worded:

“Would you say that the health of your teeth, lips, jaws and mouth is…?” with a 5-point response format ranging from ‘Excellent’ to ‘Poor’.

“How much does the condition of your teeth, lips, jaws or mouth affect your life overall?” with a response range from “Not at all” to “Very much”.

These questions are routinely used with the PPQ [26], and have been included in several UK studies [27].

Using a child-centred qualitative approach with participatory activities, children’s experiences and the acceptability of the three caries management strategies to children will be explored towards the end of trial [28].

#### *Economic*

To allow a full understanding of cost-effectiveness and add value to the analysis, two different ways of measuring incremental costs will be compared; a time/material-based cost and the current cost to the NHS.

Time and material-based cost: an appropriate fee structure and an understanding of the opportunity costs will be essential prior to implementation of any arm of the trial. It is known that fee structures influence practice. However, they do not necessarily represent the costs related to the dentist’s time and materials and may result in perverse incentives. Furthermore, there is no specific fee for some of the procedures encompassed in the biological arm, despite different time and material costs. Consequently, a “procedure cost” based on time in the surgery and materials used will be applied for the common operative interventions in the Conventional with Prevention, Biological with Prevention and Prevention Alone arms. Data on resource use will be collected via the CRF for each enrolled patient for every scheduled and unscheduled visit, and include the number of dental visits, treatments undertaken and appointment duration. These data will be combined with a micro-costing study based on data recorded from direct observation of a number of centres during the trial. The micro-costing study will estimate the resources used to provide the interventions, e.g. equipment (disposable and reusable) consumables and staff mix. The costs of onward referral (for example, for hospital admission for extraction of painful teeth under general anaesthesia) will be obtained from existing data available within the NHS.

Current cost to the NHS: the payment systems in Scotland and England/Wales differ, therefore the costs of clinical interventions to the NHS will be calculated using the standard fees from the fee per item arrangements in Scotland and an agreed Unit of Dental Activity (UDA) value in England/Wales. The UDA information will be collected annually via a short survey sent to each participating practice. In the event that this information cannot be collected from practices, the PCT or equivalent body will be asked to provide this information. Fee per item information will be based on nationally available data (Information Services Division, Scotland). This costing strategy will allow actual NHS costs to be calculated whilst highlighting any variability in cost effectiveness resulting from the different payment systems.

Data on parental costs (time off work, child care costs and over-the-counter medications) will be collected using previously developed questionnaires. These questionnaires will be completed by the accompanying adult each time a child visits the dentist.

Cost-effectiveness analysis: the relative cost-effectiveness of each arm will be assessed by considering both the cost per infection-free patient and cost per pain-free patient. The incremental cost per pain/infection episode will be calculated, with usual dental care (conventional caries management) as the base case. Sensitivity analysis will be performed to test the robustness of the results to realistic variations in the levels of the underlying data.

#### *Acceptability of treatment strategies and experiences of patients and parents*

To measure the acceptability of the treatment strategies experienced, dental anxiety of children will be assessed. The Modified Child Dental Anxiety Scale - faces (MCDASf) is a rating scale based on faces instead of the original numeric form. The reliability and validity of MCDASf has previously been evaluated for use in children in the UK [29]. The MCDASf will be administered at baseline and every recall and treatment appointment to provide information on children’s perceptions of each dental experience throughout the study.

At the start of each appointment the child will be given a faces-based Visual Analogue Scale (VAS) to report on their level of anxiety prior to arriving at the dentist’s for their appointment. They will also be given a “faces” VAS following each treatment appointment to report on their level of anxiety during treatment.

Parents’ assessment of their child’s anxiety level prior to arrival at the dentist’s for their appointment and following treatment will also be recorded using a VAS.

Given the difficulty in measuring children’s attitudes towards treatment strategies, identified in the pilot rehearsal trial, the acceptability of the three treatment strategies will be explored using child-centred interviews which incorporate child participatory activities to allow children rather than adults to shape the data collection process [28].

Discomfort during dental treatment will be assessed using a VAS – completed by the child. A VAS is often used with children to assess self-reporting of such measures as fear or pain and can be used from a very young age with acceptable levels of reliability [24]. At the end of each appointment the child will be given a faces VAS to report on their levels of pain in relation to that particular visit. In addition, parents will also be asked to report on their perceptions of their child’s levels of pain regarding that particular visit to the dentist.

#### *Dentists’ preferences*

Exploration of dentists’ preferences between the 3 treatment strategies will be explored qualitatively through interviews/focus group using a method most convenient to study dentists, using topic guides derived from qualitative information collected during the FiCTION pilot rehearsal study.

### Data management and statistical methods

#### *Data management*

To preserve confidentiality, all patients will be allocated a unique study identifier, which will be used on all data collection forms and questionnaires; names or addresses will not appear on completed questionnaires or case report forms. Only a limited number of members of the research team will be able to link this identifier to patient-identifiable details (name & address) which will be held on a password- protected database. The Chief Investigators will preserve the confidentiality of participants in the study and the Sponsor organisation (University of Dundee) will ensure that the study complies with the Data Protection Act 1998 and that all investigators and staff involved with the study comply with its requirements with regard to the collection, storage, processing and disclosure of personal information uphold the Act’s core principles. Published results will not contain any personal data that could allow identification of individual participants.

#### *Statistical methods*

The primary outcome, the proportion of children reporting either pain or sepsis during the three year follow up period, will be analysed using a mixed model with a binomial error structure. The dependent variables will be a binary indicator of pain or sepsis; variation between strata (dental practices) will be fitted as a random effect; differences between study treatments will be fitted as fixed effects. Estimates of the relative risk of pain or sepsis in the three groups will be presented in the form of odds ratios and associated 95% confidence intervals. As secondary analysis of the primary outcome, pain and sepsis will be analysed separately using the same approach.

Secondary outcomes will be analysed using multilevel modelling (repeated measures nested within children nested within general dental practices) with an appropriate error structure (binomial for binary variables, normal for continuous variables). Variation between dental practices, variation between children and variation between occasions will be modelled as random effects; difference between groups will be included as fixed effects. Within this framework we will be able to estimate:

1. The mean difference between groups at the end of the follow up period;

2. The mean difference between groups across the whole of the follow up period; and

3. The difference in the rate of change of the outcome across the follow up period

For each outcome the primary comparison of interest will be specified in the statistical analysis plan which will be finalised prior to completion of data collection.

Economic analysis will use estimates of costs and effects estimated for each trial participant to calculate the incremental cost-effectiveness ratios for the follow-up period. Where appropriate the analysis will mirror the statistical analysis. The perspective of the analysis will be the patient and the care provider. The results of the analyses will be presented as point estimates of mean incremental costs and effects. Sensitivity analysis will be used to assess the robustness of the results to realistic variations in the levels of the underlying data. In addition, techniques such as bootstrapping will be used alongside sensitivity analysis to address uncertainty. Data will be presented as cost-effectiveness acceptability curves.

### Trial management and monitoring

#### *Adverse event reporting & investigator responsibilities*

Table 2 contains a breakdown of the common & well understood consequences of treatment, less common and unpleasant side-effects and rare events associated with the techniques used in FiCTION. Investigators will report all Adverse Events on discovery to the NCTU.

**Table 2:** Adverse events which include common & well understood consequences of treatment, less common and unpleasant side-effects and rare events

	**Adverse event**		
**Procedure**	**Common & well understood consequences of treatment**	**Less common & unpleasant side effects**	**Rare events**
**Restorations in teeth and crowns on teeth (conventional)**	● Occlusal discomfort	● pain, pulpitis	● trauma to soft tissues
	● damage to adjacent teeth	● localised reaction to bonding agents or restoration materials	
	● caries progression	● dental abscess	
		● facial swelling	
**Crowns on teeth (Hall)**	● immediate gingival discomfort/ pain	● longer lasting gingival pain	● localised reaction to crowns
	● occlusal discomfort	● pulpitis	
		● dental abscess	
		● facial swelling	
**Inhalational sedation**		● dizziness and nausea	● under- or over-sedation
**Local anaesthetic**	● pain at site of injection (during or immediately following injection)	● self-inflicted trauma to soft tissues	● trismus
			● prolonged altered sensation
			● swelling
			● haematoma
			● allergic reaction
**Extraction of tooth**	● pain around site	● early and delayed post extraction bleeding	● TMJ pain
	● swelling	● infection of socket	
**Fluoride varnish**			● nausea post-application
			● allergic reaction
**Fissure sealants**		● caries progression	
**Acid etch on teeth prior to restoration or fissure sealant**		● discomfort and minor irritation of oral tissues	

#### *Trial recruitment monitoring*

Recruitment and retention rates will be monitored by the Trial Manager in the NCTU and reported at Data Monitoring and Ethics Committee and at Trial Steering Committee meetings. In addition, reports will be sent to the HTA every 2 months.

### Dissemination of results and publication policy

The results of the study will be published as a monograph for the National Institute for Health Research (NIHR) HTA, and as research papers in academic journals.

All dentists and patients participating in the trial will be given access to a summary of the trial findings via the trial website after the final HTA report is prepared.

### Trial status

The FiCTION Trial is open for recruitment of patients with the date for complete enrolment (n = 1461) being projected as the end of June 2013.

## Discussion

Dental caries is the most common disease of childhood, with a large health and economic impact. The FiCTION Trial is an NIHR HTA funded trial being undertaken across the UK to help address deficiencies in the evidence for management of dental caries in children. As a pragmatic parallel group, patient-randomised trial set in general dental practice, FiCTION aims to eradicate the uncertainty that exists among dental practitioners when treating and managing caries in children’s primary teeth. By providing evidence for the most clinically-effective and cost-effective approach to managing caries in children’s primary teeth in Primary Care, general dental practitioners will be supported in treatment decision making for child patients to minimize pain and infection in primary teeth.

In order to ensure the results of this trial are widely applicable, the geographical areas that are included in the FiCTION Trial have been selected to yield a cross-section of practices, operating in a range of different environments and circumstances (e.g. high, middle or low income communities, fluoridation status, ethnic mix, method of remuneration of GDPs (capitation and fee for item of service or a banded payment system based on Units of Dental Activity (UDA)).

The study team is multidisciplinary and broad-based, involving half of the UK Dental Schools, as well as acknowledged experts in other relevant fields. This will ensure that whilst the trial design and conduct is of the highest standard, it remains practical and pragmatic at all times. The experience of undertaking the FiCTION Pilot Rehearsal Trial and the parallel FiCTION Feasibility Study (Protocol ID HTA Project 07/44/03 NCTU:FS77044005) was beneficial with the resultant minor refinements to the design and conduct of the main trial already being appreciated. This includes changes to the presentation of parent and child information and streamlining of the recruiting process [15]. We are now confident in being able to recruit the target number of dental practices but expect that the timescale for recruiting the required number of 3–7 year old participants will be challenging. The pilot rehearsal trial confirmed that most eligible children and their parents are willing to participate, however the inclusion criterion of untreated caries requires dentists to screen a significant number of patients.

We expect the FiCTION Trial to provide evidence that will benefit the future dental care of children, improve outcomes of treatment and inform decision making by policy makers, clinicians and patients, within and outwith the UK National Health Service.

## Abbreviations

CPD: Continuing professional development; CRF: Case report form; CLRN: Comprehensive local research network; DDQ8: Dental discomfort questionnaire; GDP: General dental practitioner; HTA: Health technology assessment; ICDAS: International caries detection and assessment system; MCDASf: Modified child dental anxiety scale; NHS: National health service; NCTU: Newcastle clinical trials unit; NIHR: National institute for health research; PCT: Primary care trust; PPQ: Parents’ perception questionnaire; OHQoL: Oral health related quality of life; PCRN: Primary care research network; PMC: Preformed metal crown; RCT: Randomised control trial; UDA: Unit of dental activity; VAS: Visual analogue scale.

## Competing interests

All authors declare: no support from any organisation for the submitted work; no financial relationships with any organisations that might have an interest in the submitted work in the previous 3 years; no other relationships or activities that could appear to have influenced the submitted work.

## Authors’ contributions

Principal responsibility for study design, conduct and project management is assumed by NI, JC, GD and AM as Joint CIs for the study and CS as the Senior Trials Manager. NI and GD conceived the research and together with BC, JC, CD, MD, DE, RF, AM, EM, NP, HR, NP, CS, JS, FW designed the initial pilot trial. Subsequent design changes for this main trial were informed by ZM. All authors were involved in the development of the trial protocol. BC, CD, DE, NI, AM and FW wrote the clinical procedure manuals, NI and AM wrote the manuscript and JC, CS and GD made contributions and critical revisions to the manuscript. All authors have read, commented critically and approved the final manuscript.

## Pre-publication history

The pre-publication history for this paper can be accessed here:

http://www.biomedcentral.com/1472-6831/13/25/prepub

## ^The FiCTION trial collaboration consists of

**Co-Chief Investigators** – NPT Innes (NI)^1^, JE Clarkson (JC)^1^, GVA Douglas (GD)^2^ and A Maguire (AM)^3^,

**Additional Clinical Leads** – B Chadwick (BC)^4^, C Deery (CD)^5^, M Duggal (MDu)^2^, NI, AM, Ferranti Wong (FW)^6^

**Core Trial Management** – C Speed (CS)^7^, M Palmer (MP)^7^ and A Keightley (AK)^1^

**Grant Holders** – GD, NI, BC, JC, CD, M Deverill (MDe)^7^, MD, DJP Evans^1^, R Freeman^1^, E McColl (EM)^7^, AM, NB Pitts (NP)^8^, I.N. Steen (INS)^7^

**Additional Collaborators** – I Chestnut^4^, S Fayle^2^ , C Longbottom^8^ , Z Marshman^5^ H Rodd^5^, J Steele^3^, E Treasure^4^, R Welbury^9^

**Project Management** – JC, GD, NI, AM, CS, MP, AK, M Laird^1^, A Caldwell-Nichols^1^, E Barker^1^, S Thompson^3^, B Philpott^2^, B Howell^4^, S Lais^6^, S O’Rourke^7^, J Mills-Ball^7^, L Ternent^7^, V Ryan^7^, MD, EM

^1^Dundee Dental School, University of Dundee, Park Place, DD1 4HN, UK.

^2^Dental Institute, Leeds University, UK.

^3^Centre for Oral Health Research, School of Dental Sciences, Newcastle University, UK.

^4^Cardiff School of Dentistry, College of Biomedical and Life Sciences, Cardiff University, UK.

^5^Sheffield Unit of Oral Health & Development, School of Clinical Dentistry, University of Sheffield, UK.

^6^Institute of Dentistry, Queen Mary University of London, UK.

^7^Newcastle Clinical Trials Unit, Institute of Health and Society, Newcastle University, UK.

^8^Dental Institute, King’s College London, UK.

^9^Glasgow Dental Hospital & School, University of Glasgow, 378 Sauchiehall Street, Glasgow, UK.
